# Oral Administration of *Lactiplantibacillus plantarum* KBL396 Regulates Serum 5-Hydroxytryptamine and Gut Microbiota: Evidence from a Preclinical Mouse Model and a Randomized Controlled Human Trial

**DOI:** 10.1007/s12602-025-10811-z

**Published:** 2025-11-17

**Authors:** Woojae Myung, Sung Jae Jang, Giljae Lee, Cheonghoon Lee, Kiuk Lee, Sung Hyun Moon, Yunsun Jeong, Woon-Ki Kim, SungJun Park, Hyungjin Lee, Yun Seong Park, Sangah Shin, Tae-Wook Nam, Hong Jin Jeon, GwangPyo Ko

**Affiliations:** 1 Department of Neuropsychiatry, Seoul National University Bundang Hospital, Gyeonggi-Do, Republic of Korea; 2 Department of Psychiatry, Seoul National University College of Medicine, Seoul, Republic of Korea; 3 Department of Environmental Health Sciences, Graduate School of Public Health, Seoul National University, Seoul, Republic of Korea; 4 weBiom Inc., Seoul, Republic of Korea; 5 Institute of Health and Environment, Seoul National University, Seoul, Republic of Korea; 6 KoBioLabs, Inc, Seoul, Republic of Korea; 7 N-Bio, Seoul National University, Seoul, Republic of Korea; 8 Department of Food and Nutrition, Chung-Ang University, Gyeonggi-Do, Republic of Korea; 9 Department of Psychiatry, Depression Center, Samsung Medical Center, Sungkyunkwan University School of Medicine, Seoul, Republic of Korea; 10 Digital Therapeutics Research Center, Samsung Medical Center, Seoul, Republic of Korea; 11 Department of Health Science of Clinical Research of Medical Device Management & Research, and Department of Clinical Research Design & Evaluation, Samsung Advanced Institute for Health Sciences & Technology (SAIHST), Sungkyunkwan University, Seoul, South Korea

**Keywords:** 5-Hydroxytryptamine, Chronic social defeat stress, Gut–brain axis, *Lactiplantibacillus plantarum*, Probiotic

## Abstract

Emerging evidence links gut microbiota to major depressive disorder via the gut–brain axis. Here, we provide comprehensive evidence from a preclinical mouse model and a randomized controlled human trial for the psychobiotic potential of *Lactiplantibacillus plantarum* (*L. plantarum*) KBL396, a strain isolated from a healthy Korean adult. We demonstrate that *L. plantarum* KBL396 potently induces *Tph1* gene expression in vitro (*p* < 0.001), which encodes the rate-limiting enzyme tryptophan hydroxylase 1 for 5-hydroxytryptamin (5-HT) synthesis. *L. plantarum* KBL396 administration ameliorated depressive-like behaviors (*p* < 0.05), increased serum 5-HT, and modulated immune cell populations in mice with chronic social defeat stress. Critically, these preclinical findings translated to humans: the 8 weeks of randomized, double-blind, placebo-controlled trial (the KBL396 group: *n* = 62; the placebo group: *n* = 30) showed that *L. plantarum* KBL396 significantly elevated the serum 5-HT and dopamine level (*p* < 0.05). *L. plantarum* KBL396 administration remodeled gut microbiota with the increases of *Lactiplantibacillus* and *Akkermansia*. Collectively, our findings preliminarily demonstrate that *L. plantarum* KBL396 is a psychobiotic candidate that mechanically links gut-derived neurotransmitter production to beneficial mental health-related outcomes across preclinical model and clinical settings.

**Graphical Abstract:**

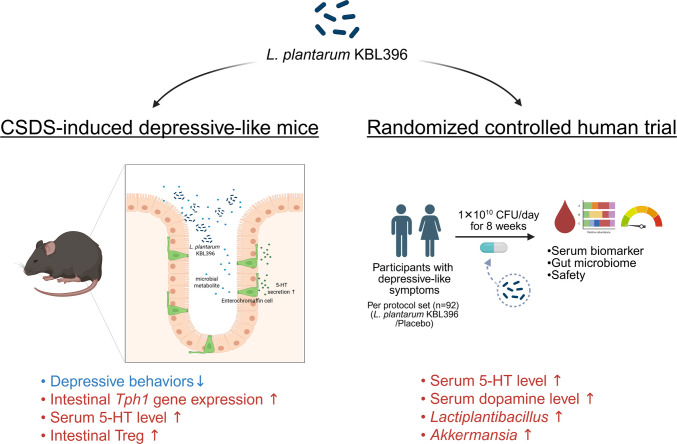

## Introduction

The gut microbiota plays a fundamental role in regulating host physiology, including immune function, metabolic processes, and neural signaling [1]. Recent studies have highlighted that microbial metabolites, such as short-chain fatty acids and amino acid derivatives, mediate communication along the microbiota–gut–brain axis, a bidirectional network connecting the gastrointestinal tract and the central nervous system (CNS) [1]. This interaction is increasingly recognized as a key factor in maintaining neuroimmune and behavioral homeostasis.

Accumulating evidence suggests that dysbiosis of gut microbiota is associated with neuropsychiatric conditions. For example, patients with major depressive disorder (MDD) exhibit distinct alterations in the gut microbial composition [2–7]. In a large human study, 13 microbial taxa linked to the synthesis of glutamate, butyrate, 5-hydroxytryptamine (5-HT), and gamma-aminobutyric acid (GABA) showed negative associations with depressive symptoms [2]. Moreover, the abundances of *Coprococcus* and *Faecalibacterium* spp. were positively correlated with quality-of-life indicators, whereas *Dialister* and *Coprococcus* spp. were significantly reduced in individuals with MDD [3].

Experimental data support these associations. Transplantation of the fecal microbiota from patients with MDD into mice can induce depression-like behavioral phenotypes [8–11]. Such dysbiosis has been linked to neuroinflammation, hypothalamic–pituitary–adrenal axis dysregulation, impaired neurogenesis, and altered neurotransmitter profiles [8, 12–15]. Germ-free mice demonstrate elevated corticosterone levels and reduced serum 5-HT, underscoring the influence of the microbiota on stress-related neurochemistry [16, 17].

Among gut-derived neuroactive molecules, 5-HT and dopamine are particularly importance. Approximately 90% of the human body’s 5-HT is produced in the gut, where the microbiota is a key regulator of its synthesis [18]. The gut microbiota also contributes to over 50% of peripheral dopamine production, a catecholamine crucial for maintaining mood and immune responses [19]. Accordingly, the preclinical evidence has been suggested that various *Lactobacillus*, such as *Lactiplantibacillus plantarum* (*L. plantarum*) and *Lactocaseibacillus rhamnosus*, can successfully attenuate anxiety and depressive-like behaviors [20, 21].

Therefore, this study investigated the psychobiotics potential of *L. plantarum* KBL396, a novel strain from a healthy Korean adult, using both a preclinical mouse model and a randomized controlled human trial. First, we used mice with chronic social defeat stress (CSDS) to evaluate the effect of *L. plantarum* KBL396 on behavioral outcome, serum 5-HT level, and immune modulation. Subsequently, we conducted a multi-center, randomized, double-blinded, placebo-controlled trial assessing changes in serum 5-HT and dopamine concentrations, as well as gut microbiota composition, following the 8 weeks of *L. plantarum* KBL396 administration. This integrated design provides a robust evaluation of *L. plantarum* KBL396 for supporting mental health via the gut–brain axis.

## Materials and Methods

### Preparation of Bacteria

Forty-seven lactic acid bacteria (LAB) strains—19 strains of *Lactobacillus* spp., including *L. plantarum* KBL396, and 28 strains of *Bifidobacterium* spp.—were isolated from the feces of healthy Korean adults and cultured anaerobically at 37 °C for 24 h using Lactobacilli MRS broth (Becton, Dickinson and Company, Sparks, MD, USA) with 0.05% L-cysteine hydrochloride and an AnaeroPack (Mitsubishi Gas Chemical Company, Tokyo, Japan), as described previously with some modifications [22]. For further in vitro and in vivo CSDS models, bacterial cells were harvested via centrifugation at 13,000 rpm for 5 min and stored at 4 °C until use. The supernatant was neutralized to pH 7.0 using 1 N NaOH and then filtered through 0.22-μm Millex-GV syringe filter units (Merck Millipore, Cork, Ireland) and stored at − 20 °C until use.

For the human study, lyophilized *L. plantarum* KBL396 powder was produced by LactoMason (Gyeongsangnam-do, Republic of Korea) and encapsulated by PioPharm (Gyeonggi-do, Republic of Korea). Placebo capsules, primarily composed of maltodextrin, were manufactured to match the appearance, flavor, and color of *L. plantarum* KBL396 capsules. Bacterial concentrations were determined using the cultivation method and expressed as colony-forming units (CFUs). All capsules were stored at 2–8 °C until use.

### Analysis of *Tph1* Gene Expression in an In Vitro Model

RIN-14B cells (CRL-2059) were purchased from the American Type Culture Collection and cultured at a density of 4 × 10^5^ cells per well in 24-well plates at 37 °C with 5% CO_2_ for 24 h in 0.5 mL RPMI 1640 medium (Thermo Fisher Scientific, Waltham, MA, USA) containing 10% fetal bovine serum (Thermo Fisher Scientific), 100 U/mL penicillin (Thermo Fisher Scientific), and 100 μg/mL streptomycin (Thermo Fisher Scientific), as described previously [23]. Subsequently, the medium was replaced with 0.4 mL supernatants derived from LAB strains, followed by incubation at 37 °C for 1 h. Lactobacilli MRS broth was used as a negative control, while Lactobacilli MRS broth with 250 µmol/L deoxycholate was used as a positive control.

Changes in *Tph1* gene expression in RIN-14B cells were assessed using quantitative real-time polymerase chain reaction (qRT-PCR). Briefly, total RNA of RIN-14B cells was extracted using an easy-spin Total RNA Extraction Kit (iNtRON Biotechnology, Gyeonggi-do, Republic of Korea) and reverse-transcribed using a High-Capacity RNA-to-cDNA kit (Thermo Fisher Scientific), according to the manufacturer’s instructions. qRT-PCR was performed using a QuantStudio 5 Real-Time PCR System (Thermo Fisher Scientific), a Power SYBR Green PCR Master Mix (Thermo Fisher Scientific), and 0.01 mmol/L primers (*Tph-1* forward: 5′—GGC TTT GAG GTC CTC TTT CCA—3′, *Tph-1* Reverse: 5′—CCC CCT TTC TGA GGA ATG GTC—3′, *Gapdh* forward: 5′—AAC TTT GGC ATT GTG GAA GG—3′, *Gapdh* reverse: 5′—GGA TGC AGG GAT GAT GTT CT—3′) under the following conditions: denaturation at 95 °C for 10 min, followed by 40 cycles of 95 °C for 15 s and 60 °C for 60 s. The *Tph1* gene expression was normalized using glyceraldehyde-3-phosphate dehydrogenase.

### Analysis of Acetic Acid and Lactic Acid Production

*L. plantarum* KBL396 was cultured anaerobically at 37 °C for 12, 18, 22, 24, and 36 h in Lactobacilli MRS broth (Becton, Dickinson and Company). The supernatant was collected via centrifugation at 13,000 rpm for 5 min and filtered using 0.22-μm Millex-GV syringe filter units (Merck Millipore). Subsequently, internal standards (1% 2-methylpentanoic acid for acetic acid and benzoic acid for lactic acid) and extraction solvents (ethyl ether for acetic acid and chloroform for lactic acid) were added, as described previously with some modifications [22]. The organic layer was analyzed using an Agilent 7890A gas chromatograph (Agilent Technologies, Santa Clara, CA, USA) under the following conditions: 1.5-kV capillary voltage, 600 L/h of desolvation gas flow, 50 L/h cone gas flow, 170 °C oven temperature, and 225 °C for a flame ionization detector and an injection port temperature.

### Genomic Analysis for *L. plantarum* KBL396

First, the bacterial genome of *L. plantarum* KBL396 was extracted using a Wizard HMW DNA Extraction Kit (Promega, Madison, WI, USA). The whole genome sequencing of *L. plantarum* KBL396 was performed using a PacBio single-molecule real-time sequencing with the PacBio RS II system (Pacific Biosciences, Menlo Park, CA, USA). Prokka v. 1.14.5 (https://github.com/tseemann/prokka) [24] with eggNOG-mapper v. 2.1.12 (http://eggnog-mapper.embl.de/) [25] was used for genome annotation. Functional categorization of protein-coding genes and prediction of metabolic pathways were performed using the Clusters of Orthologous Genes (COG) database [26] and the Kyoto Encyclopedia of Genes and Genomes (KEGG) orthologous gene family database (Kanehisa Laboratories, Kyoto University; https://www.genome.jp/kegg/) [27].

### Mouse Model of CSDS

All animal experimental procedures were approved by the Institutional Animal Care of Use Committee (IACUC) of Seoul National University (Case Number: SNU-170920–10-2). Three-week-old male C57BL/6 mice and 12-week-old male CD-1 mice (Orient Bio, Gyeonggi-do, Republic of Korea) were purchased. C57BL/6 mice were divided into four groups (*n* = 10) and housed under the specific pathogen-free conditions with a 12-h light/dark cycle. All mice had free access to sterilized food and water. C57BL/6 mice were acclimated for 1 week before the experiments. Prior to the experiments, a screening process was performed to select CD-1 mice with aggressive behaviors.

The CSDS model was established as described previously with some modifications [28]. First, all C57BL/6 mice were placed with an aggressive CD-1 resident mouse for 5 min daily 7 consecutive days. Following this defeat session, each C57BL/6 mouse was housed with an aggressive CD-1 resident mouse in the same cage for 24 h. A transparent acrylic divider was installed in the middle of cage to separate C57BL/6 mice from the aggressive CD-1 resident mouse, while still maintaining psychosocial stress throughout the experiments. The CD-1 mice paired with a C57BL/6 mouse were rotated daily to prevent habituation. C57BL/6 mice housed with another C57BL/6 mouse were used as the control group. *L. plantarum* KBL396 (approximately 1 × 10^9^ CFUs) was administered orally daily via drinking water from day 28 before CSDS induction until the end of the experiments (~ 7 days). After 7 days of CSDS induction, behavioral tests were performed as described below.

### Spontaneous Alteration Test (SAT)

The SAT was performed using an acrylic three-arm (3-cm wide × 36-cm deep × 15-cm high) Y-maze [29]. First, mice were left to adapt for 30 min in the Y-maze before being placed in one arm. Subsequently, they were allowed to explore freely for 8 min. The number of arm entries was recorded, and the spontaneous alteration percentage was calculated as follows: Spontaneous alteration (%) = [(number of alterations)/(number of total entries − 2)] × 100.

### Social Interaction Test (SIT) [28]

Initially, mice explored a white open field chamber (42-cm wide × 42-cm deep × 42-cm high) for 3 min, which was divided into corner zones and an interaction zone (a circular grid cage, 10-cm diameter × 10-cm high). Then, an aggressive CD-1 mouse was placed in the interaction zone and changes in the mouse travel patterns were assessed for 3 min. The total time spent in corner zones and the interaction zone were measured using an EthoVision XT software (Noldus, Leesburg, VA, USA). The social interaction (SI) ratio was calculated as follows: SI ratio = (Time spent in the interaction zone with an aggressive CD-1 mouse)/(Time spent in the interaction zone without an aggressive CD-1 mouse).

### Tail Suspension Test (TST)[30]

Mice were suspended from a horizontal bar (30 cm above the ground) for 6 min. Adhesive tape was used to attach the tip of the tail (~ 2 cm) to the bar. Simultaneously, the total immobile time of mice, i.e., the period of complete motionless of all limbs, was measured. After completing all behavioral tests, the mice were euthanized, and blood, colon, and mesenteric lymph node (MLN) tissues were collected for further analyses.

### Enzyme-Linked Immunosorbent Assay (ELISA) and Flow Cytometry

Collected blood samples were inverted several times and incubated at room temperature for 30 min. Serum was separated via centrifugation at 1800 rpm for 5 min at 4 °C and stored at − 80 °C until use. Then, serum 5-HT levels were measured using a Serotonin Ultrasensitive ELISA Assay Kit (Eagle Bioscience, Amherst, NH, USA), according to the manufacturer’s instructions. T cells and dendritic cells (DCs) from MLN tissues were collected as described previously [31]. Briefly, MLN tissues were gently dissociated and filtered using a cell stainer (pore size = 100 μm). Cells were subjected to Fc gamma receptor blockade and surface stained for 30 min at 4 °C. To assess cell viability, a Fixable Viability Stain 510 (FVS510, BD Biosciences, San Jose, CA, USA) was applied to cells. Subsequently, isolated T cells were stained with CD3^+^ fluorescein isothiocyanate (BD Bioscience), CD4^+^ PerCP/Cyanine5.5 (BD Bioscience), and CD25^+^ phycoerythrin (BD Bioscience) according to the manufacturer’s instructions. Intracellular staining was performed using Foxp3^+^ Alexa Flour 647 (BD Bioscience) according to manufacturer’s instructions. Isolated DCs were stained with CD11c^+^ phycoerythrin (BD Bioscience) according to the manufacturer’s instructions. Flow cytometric analyses were performed using a BD FACSVerse Flow Cytometer (BD Bioscience) and the data were analyzed with FlowJo Software (BD Bioscience).

### Multi-center, Randomized, Double-Blind, Placebo-Controlled Human Study

The human study was conducted in accordance with the Korean Good Clinical Practice (KGCP) Guidelines and the Declaration of Helsinki. All participants provided informed consent, and the study protocol was approved by the institutional review boards of Samsung Medical Center (No. SMC 2021–02–150–003) and Seoul National University Bundang Hospital (No. B-2013/673–006).

A total of 193 individuals visited the study sites between July 2021 and November 2022, and 105 participants met the following inclusion criteria (Fig. 4). The inclusion criteria are as follows: (1) aged 19 or older; (2) Korean version of the Pittsburgh Sleep Quality Index (PSQI-K) ≥ 5; (3) Stress Response Inventory (SRI) score between 81 and 120; (4) Korean-Beck Anxiety Inventory (K-BAI) score between 20 and 45 or Korean-Beck Anxiety Inventory-II (K-BDI-II) score between 20 and 45; (5) voluntary participation with written informed consent. The major exclusion criteria included the following: (1) severe illnesses such as cardiovascular, immune, respiratory, gastrointestinal, urological, and musculoskeletal, and infectious diseases and malignant tumors; (2) ongoing treatment for alcohol abuse, cardiovascular disease, or CNS disorders; (3) treatment for mental illnesses diagnosed according to the Diagnostic and Statistical Manual of Mental Disorders (DSM-5, including MDD, anxiety disorders, post-traumatic stress disorder, obsessive–compulsive disorder, schizophrenia, and bipolar disorder; (4) dementia treatment within the last 3 months; (5) treatment for sleep disorders such as insomnia, periodic limb movement disorder, restless leg syndrome, or sleep apnea within the last 4 weeks; (6) nicotine or alcohol withdrawal symptoms; and (7) any conditions deemed inappropriate by investigators.

Participants were randomly assigned to either the *L. plantarum* KBL396 or the placebo group in a 2:1 ratio (Fig. 4) using a SAS v.9.4 Software (SAS Institute, Cary, NC, USA). *L. plantarum* KBL396 or placebo capsules were provided to participants every 4 weeks based on the randomization table. Participants took six capsules daily, containing 1 × 10^10^ CFUs of *L. plantarum* KBL396, the maximum daily dosage prescribed by the Ministry of Food and Drug Safety (MFDS) of Republic of Korea, or placebo, for 8 weeks. To ensure study integrity, both investigators and participants were blinded to the allocation codes until study completion.

Demographic characteristics of participants were documented at visit 1. Lifestyle factors, including alcohol and caffeine consumption, smoking, exercise habits, positive life events, and smartphone usage, as well as medical histories and clinical characteristics were documented at visit 1 and visit 4. Dietary surveys were completed for eligible participants at visit 2 and visit 3. A Computer-Aided Nutritional analysis program (CAN)-Pro v. 5.0 (The Korean Nutrition Society; https://www.kns.or.kr/ can6.0/intro.asp) was used to track food and nutrient intake of participants.

Blood samples from participants were collected and transferred via a cold-chain process to SCL Healthcare (Gyeonggi-do, Republic of Korea), the central laboratory for this study. Serum neurotransmitter concentrations, including 5-HT and dopamine, were measured following the central laboratory’s protocols. Fecal samples were collected using DNA/RNA Shield-Fecal Collection Tubes (Zymo Research, Irvine, CA, USA) and immediately stored at − 20 °C. During visit 2 and visit 4, frozen fecal samples were collected and stored at − 70 °C until use.

### Analysis of Gut Microbiota

Fecal samples from participants were analyzed as previously described with some modifications [32]. Briefly, total bacterial DNA was extracted using a QIAamp Stool Mini Kit (Qiagen, Hilden, Germany) according to the manufacturer’s instructions. The V3–V4 region of the 16S ribosomal RNA (16S rRNA)-coding gene was amplified using the universal bacterial primers (341F/805R). An Illumina Miseq platform (Illumina, San Diego, CA, USA) with a MiSeq Regent Kit (Illumina) was used for metagenome sequencing. Trimmomatic v. 0.38 (USEDEL Lab; https://github.com/usadellab/Trimmomatic) [33] was used to trim the raw reads. Quantitative Insights into Microbial Ecology (QIIME) 2 v. 2024.5 (QIIME 2 Development Team; https://qiime2.org) [34] with the Silva v. 138.1 database (SILVA rRNA database project; https://www.arb-silva.de) [35] was used for taxonomic classification. The Divisive Amplicon Denoising Algorithm 2 was applied for demultiplexing, error correction, and chimera removal. Shannon indices or principal coordinates analysis (PCoA) plots with Bray–Curtis distances, representing alpha and beta diversities of gut microbiota, respectively, were generated using a q2-diversity plugin a qiime2R (https://github.com/jbisanz/qiime2R).

### Statistical Analysis

In vitro and in vivo experimental results are presented as means ± the standard error of the mean (SEM) from at least three independent experiments. When appropriate, Prism v. 10 (GraphPad Software, San Diego, CA, USA) was used for statistical analyses such as the Mann–Whitney *U* test, Wilcoxon rank-sum test, and one-way analysis of variance (ANOVA) with the Bonferroni post hoc test, along with data visualization.

SAS v.9.4 Software (SAS Institute) was used for statistical analyses for the per protocol dataset. Categorical and continuous variables are reported as frequencies with proportions or as means ± standard deviations (SD), respectively. For inter-group comparisons of categorical variables, the chi-square test or Fisher’s exact test was performed. For inter- or intra-group comparisons of continuous variables, the Wilcoxon rank-sum test or a paired *t*-test was performed, respectively. Analyses of covariance (ANCOVA) with adjustment of appropriate variables including body mass index (BMI), drink count per week, energy intake, energy-adjusted tryptophan intake, and exercise were performed using a SAS v.9.4 Software (SAS Institute). Statistical significance was set at *p*-value (*p*) < 0.05. Additionally, Spearman’s nonparametric correlation coefficients (*r*) between the serum 5-HT levels and SI ratios were calculated using R v. 4.1.2 (The R Project; https://www.r-project.org).

## Results

### Characteristics of *L. plantarum* KBL396

**Fig. 1:**
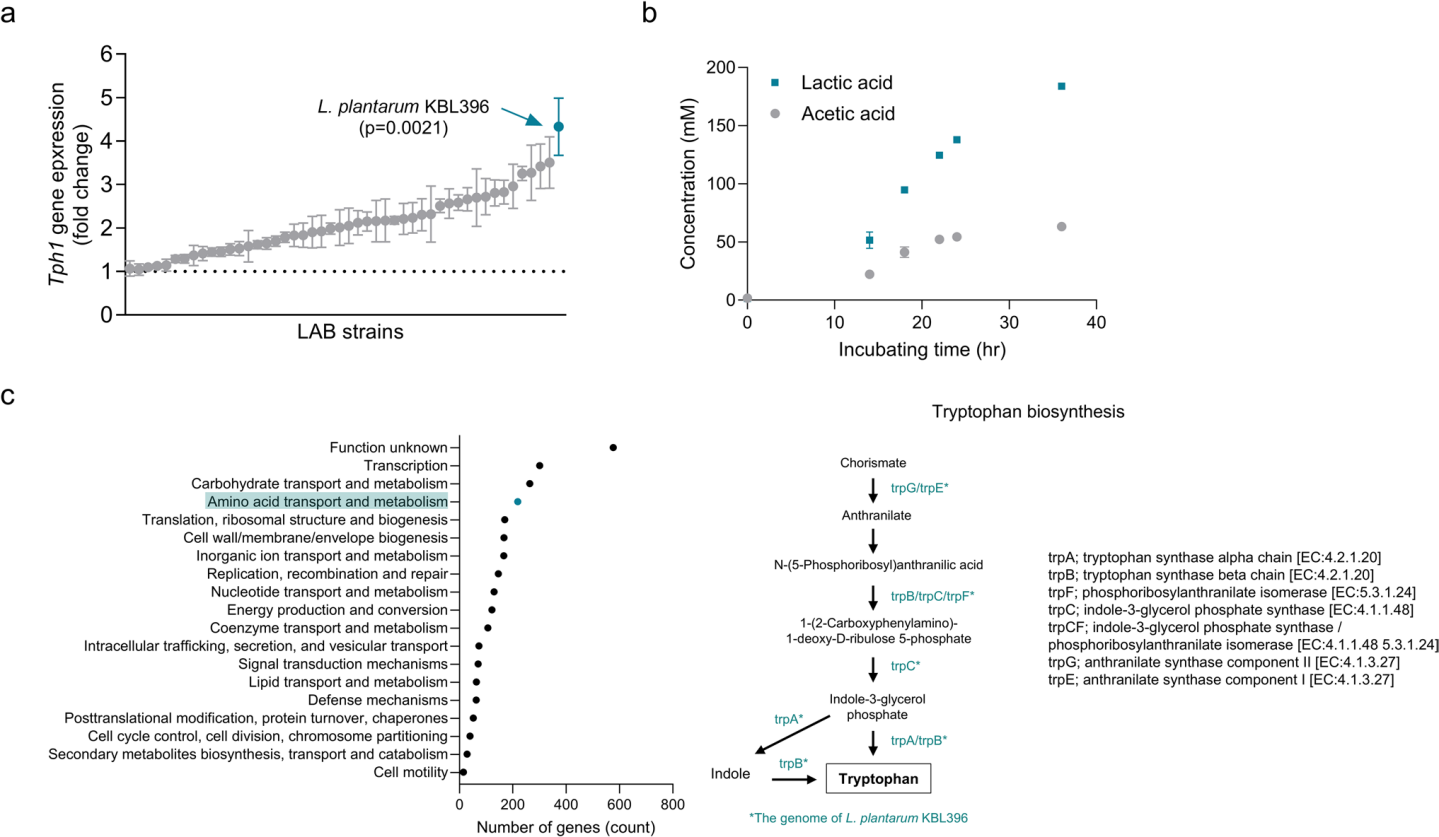
Characteristics of *L. plantarum* KBL396. **a** Effects of *L. plantarum* KBL396 on *Tph1* gene expression. **b** Acetic and lactic acid production. **c** Prediction of metabolic pathways using orthologous genes in the *L. plantarum* KBL396 genome. When appropriate, data are expressed as means ± SEM. Asterisks indicate statistical significance (***p < 0.001; one-way ANOVA with the Bonferroni post hoc test)

Supernatants derived from specific LAB strains influenced the upregulation of *Tph1* gene expression in RIN-14B cells (Fig. 1a). Among 19 strains of *Lactobacillus* spp. and 28 strains of *Bifidobacterium* spp., tested, the supernatant from *L. plantarum* KBL396 induced the highest *Tph1* gene expression (*p* < 0.001). Moreover, the lactic acid concentration in the supernatant from *L. plantarum* KBL396 increased rapidly over the cultivation period (~ 36 h) (Fig. 1b). After 18 h of cultivation, the acetic acid concentration in the supernatant reached approximately 50 nmol/L and continued to increase gradually over 36-h cultivation period.

*L plantarum* KBL396 genome contains numerous genes related to amino acid transport and metabolism (Fig. 1c). These genes were strongly correlated with predicted metabolic pathways for tryptophan metabolism, including those for such as tryptophan or indole-3-glycerol phosphate synthesis.

### Effects of *L. plantarum* KBL396 on CSDS in Mice

**Fig. 2:**
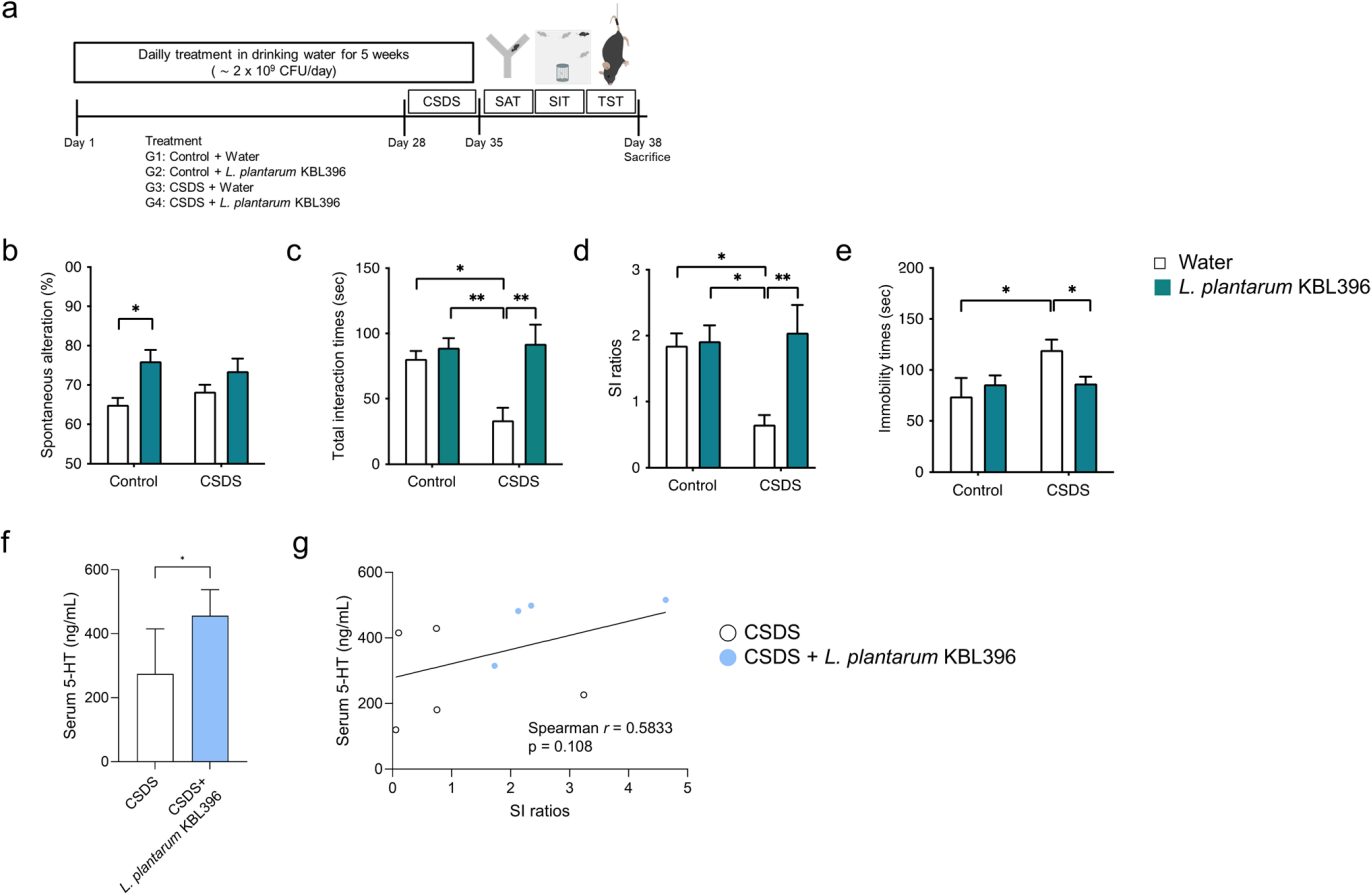
Effect of *L. plantarum* KBL396 on behavior and serum 5-HT levels in a mouse model of CSDS. **a** Experimental scheme of the CSDS model. Drinking water was used as a vehicle for oral administration of *L. plantarum* KBL396. **b** Spontaneous alteration test (SAT). **c** Total interaction time (SIT). **d** SI ratio (SIT). **e** Immobile time (TST). **f** Serum 5-HT levels. **g** Correlation between serum 5-HT levels and SI ratios. The Spearman correlation coefficient (*r*) indicates the correlation strength. When appropriate, data are expressed as means ± SEM. Asterisks indicate statistical significance (*p < 0.05; **p < 0.01; Mann–Whitney *U* test)

Figure 2a shows the experimental design of the CSDS model. In the Y-maze test, spontaneous alteration was increased in mice with CSDS + KBL396 compared to the control group (Fig. 2b). Both total interaction time (*p* < 0.01) and the social interaction (SI) ratio (*p* < 0.01) in the social interaction test were significantly higher in the CSDS + KBL396 group (Fig. 2c, d). Furthermore, in the tail suspension test, the immobility time in the *L. plantarum* KBL396-administered group was significantly shorter **(***p* < 0.05**)** than in the CSDS-only group (Fig. 2e).

Moreover, serum 5-HT levels were significantly increased in the CSDS + KBL396 group compared to the CSDS-only group (*p* < 0.05) (Fig. 2f). Interestingly, the increases in serum 5-HT levels in mice due to the *L. plantarum* KBL396 administration were positively correlated with the SI ratio (*r* = 0.5833, *p* = 0.0108) (Fig. 2g).

**Fig. 3:**
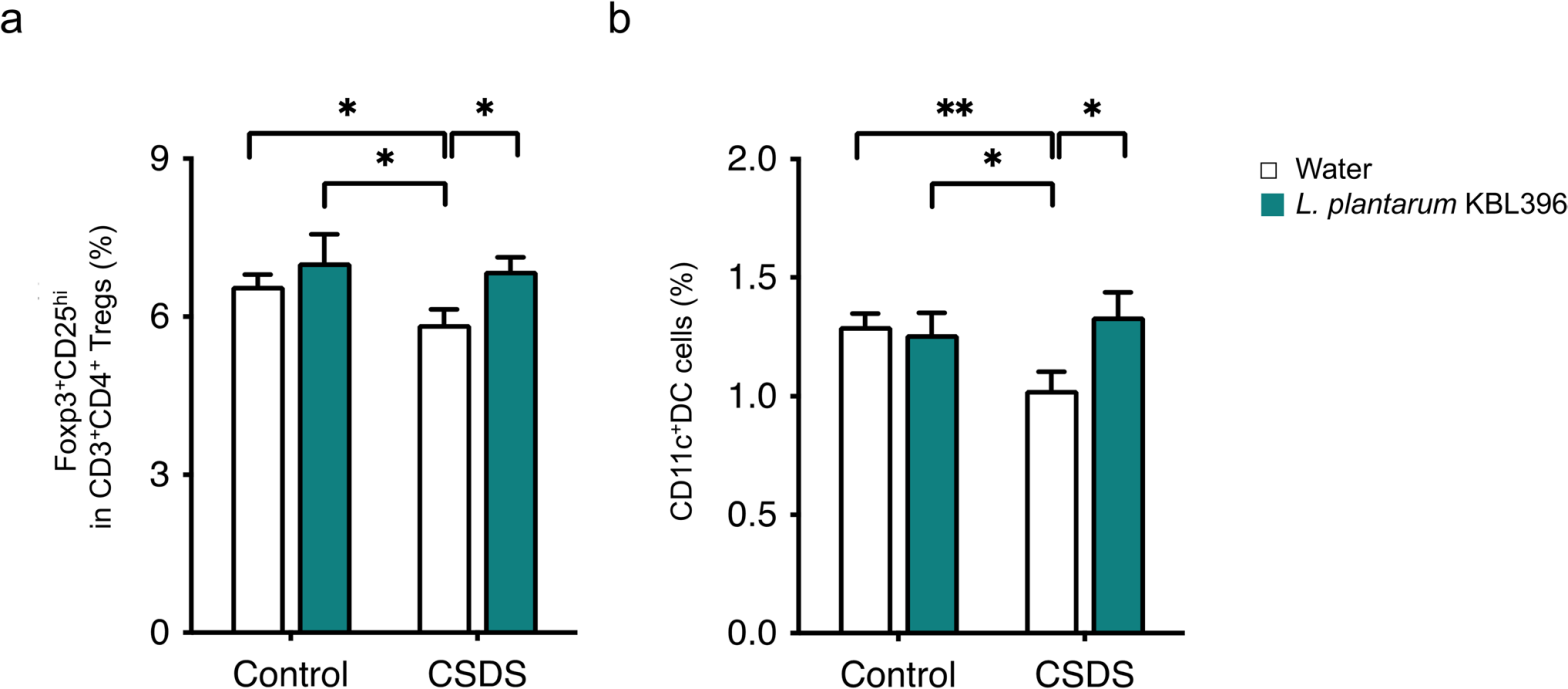
Effect of *L. plantarum* KBL396 on Tregs and DCs in a mouse model of CSDS. **a** Percentage of Foxp3^+^CD25^hi^ in CD3^+^CD4^+^ Tregs. **b** Percentage of CD11c.^+^ DCs. When appropriate, data are expressed as means ± SEM. Asterisks indicate statistical significance (*p < 0.05; **p < 0.01; Mann–Whitney *U* test)

Compared to CSDS-only mice, the percentage of Foxp3⁺CD25ʰⁱ T regulatory cells (Tregs) within the CD3⁺CD4⁺ T cell population was significantly higher in the *L. plantarum* KBL396-administered group (*p* < 0.05) (Fig. 3a). The percentage of CD11c⁺ dendritic cells (DCs) also showed a significant increase in the CSDS + KBL396 group (*p* < 0.05) (Fig. 3b).

### Demographic Characteristics and Lifestyles of Participants

**Fig. 4:**
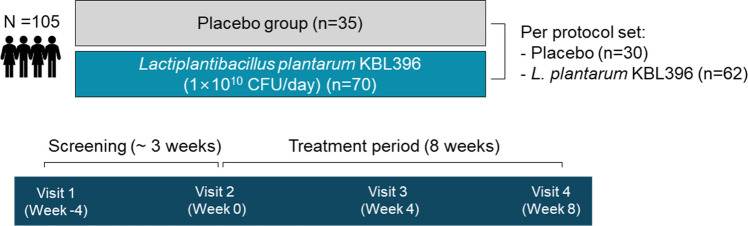
The design of the human study

A total of 92 participants were included in the per-protocol analysis: 62 participants in the *L. plantarum* KBL396 group and 30 participants in the placebo group (Fig. 4). The mean age of participants was 42.53 ± 11.25 years in the *L. plantarum* KBL396 group and 42.93 ± 12.26 years in the placebo group (Table 1). The *L. plantarum* KBL396 group consisted of nine males (14.52%) and 53 females (85.48%). The gender distribution was not significantly different from that of the placebo group (Table 1).

Key lifestyle factors, including alcohol consumption, smoking status, exercise, caffeinated coffee intake, and smartphone usage, showed no significant differences between the groups (Table 1). Furthermore, occurrences of major lifestyle factors, such as pregnancy or other positive experiences, were similar between the groups (Table 1).

**Table 1:** Demographic characteristics and lifestyle factors

Variable^1^	*L. plantarum* KBL396 group (*n* = 62)	Placebo group (*n* = 30)	*p*-value
Age (years)	42.53 ± 11.25	42.93 ± 12.26	0.8349^2^
Sex (M/F) (*n*, %)	9 (14.52)/53 (85.48)	8 (26.67)/22 (73.33)	0.1592^3^
Pregnancy			
Yes (*n*, %)	38 (71.70)	17 (77.27)	0.6192^2^
No (*n*, %)	15 (28.30)	5 (22.73)	
Alcohol consumption			0.3675^2^
Yes (*n*, %)	31 (50.00)	12 (40.00)	
No (*n*, %)	31 (50.00)	18 (60.00)	
Alcohol consumption per week (g)	22.81 ± 18.41	21.81 ± 16.16	0.9783^2^
Smoking (*n*, %)			0.0999^4^
None	61 (98.39)	27 (90.00)	
Former smoker < 1 year	0 (0.00)	1 (3.33)	
Former smoker ≥ 1 year	1 (1.26)	2 (6.67)	
Current	0 (0.00)	0 (0.00)	
Exercise per week (*n*, %)			
None	35 (56.45)	15 (50.00)	0.6669^4^
1–2	19 (30.65)	9 (30.00)	
3–4	6 (9.68)	6 (20.00)	
5–6	1 (1.61)	0 (0.00)	
Everyday	1 (1.61)	0 (0.00)	
Caffeinated coffee consumption			
Yes (*n*, %)	51 (82.26)	22 (73.33)	0.3215^3^
No (*n*, %)	11 (17.74)	8 (26.67)	
Daily coffee consumption (cups/day)	1.51 (0.80)	1.74 (1.12)	0.5630^2^
Positive life events			
Yes (*n*, %)	9 (14.52)	3 (10.00)	0.7446^4^
No (*n*, %)	53 (85.48)	27 (90.00)	
Smartphone usage time (h)/day (*n*, %)			
< 1	5 (8.06)	1 (3.33)	0.7884^4^
2–3	27 (43.55)	14 (46.67)	
4–6	21 (33.87)	11 (36.67)	
7–9	6 (9.68)	4 (13.33)	
≥ 10	3 (4.84)	0 (0.00)	

^1^Data are presented as means ± SD or number

^2^Inter-group comparisons of continuous variables were performed using a two-sample *t*-test

^3^Inter-group comparisons of categorical variables were performed using the chi-square test

^4^Inter-group comparisons of categorical variables, when sample sizes were small, were performed using Fisher’s exact test

### Effects of *L. plantarum* KBL396 on Serum 5-HT and Dopamine Levels in Participants

**Fig. 5:**
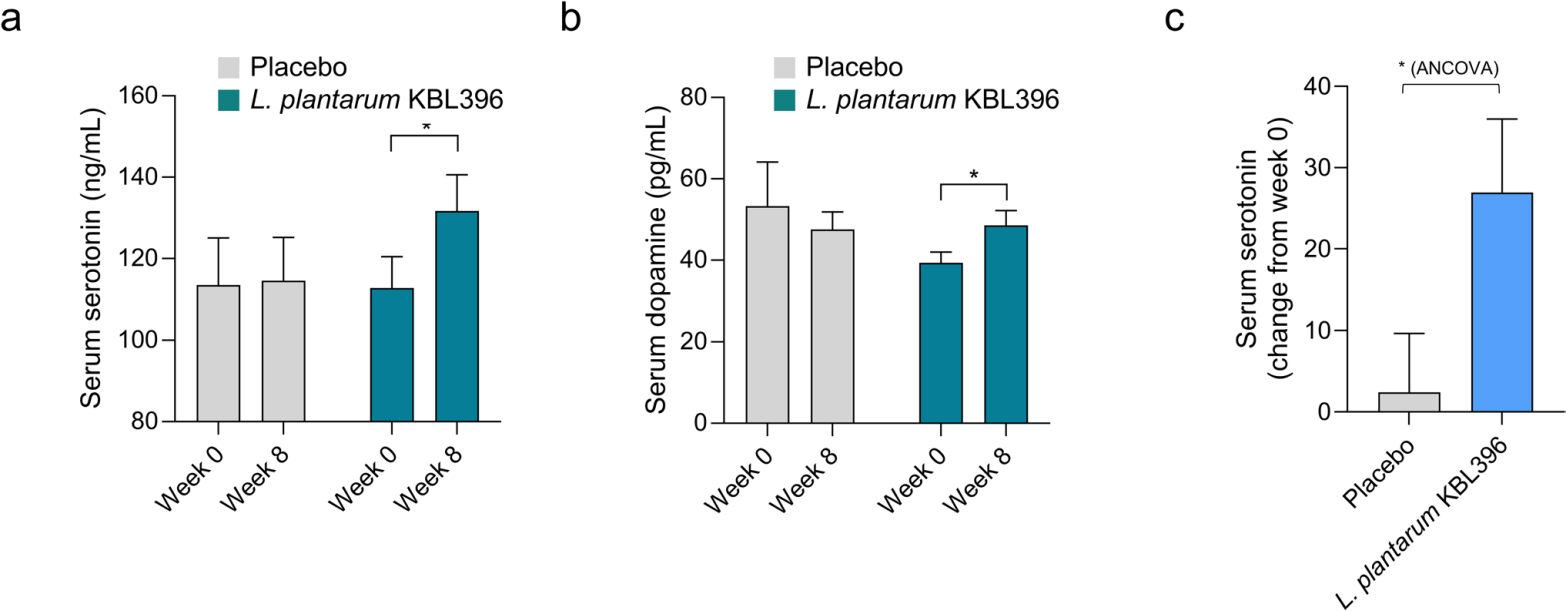
Effect of *L. plantarum* KBL396 on serum 5-HT and dopamine levels in study participants. **a** Serum 5-HT levels in the per protocol set. **b** Serum dopamine levels in the per protocol set. **c** Inter-group comparisons of changes in serum 5-HT levels from baseline to week 8 in the per protocol set. **d** Inter-group comparisons of changes in serum 5-HT levels from baseline to week 8 in participants who consumed alcohol less than 3 days per week. Asterisks indicate statistical significance (*p < 0.05; paired *t*-test or ANCOVA with adjustment of BMI, energy intake, energy-adjusted tryptophan intake, and exercise)

After 8 weeks of administration, participants in *L. plantarum* KBL396 group showed a significant increase in both serum 5-HT (*p* < 0.05) and dopamine (*p* < 0.05) levels (Fig. 5a, b). Interestingly, in participants who reported consuming alcohol less than 3 days per week, significant increases in the serum 5-HT level in the *L. plantarum* KBL396 group compared to the placebo group were discovered (*p* < *0.05*) (Fig. 5c).

### Effects of *L. plantarum* KBL396 on the Gut Microbiota in Participants

**Fig. 6:**
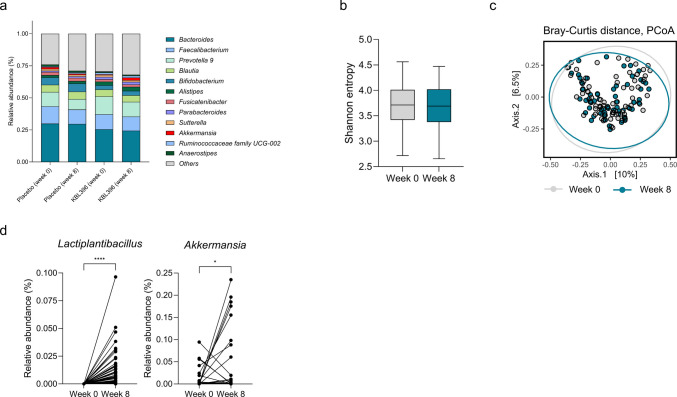
Effects of *L. plantarum* KBL396 on the gut microbiota composition in study participants in the per protocol set. **a** Taxonomic shift in the gut microbiota. **b** Alpha diversity indices in participants administered *L. plantarum* KBL396 from week 0 to week 8. . **c** PCoA plots based on Bray–Curtis dissimilarity distances in participants administered *L. plantarum *KBL396 from week 0 to week 8. **d** Changes in the relative abundances of *Lactiplantibacillus* and *Akkermansia* in participants administered *L. plantarum* KBL396 from week 0 to week 8. Asterisks indicate statistical significance (*p < 0.05; ****p < 0.0001; a paired *t*-test)

To investigate the effects of *L. plantarum* KBL396 on gut microbiota, fecal samples from participants were analyzed. At the genus level. *Bacteroides* was the most abundant taxon in both groups, while the proportion of *Bifidobacterium* differed between the *L. plantarum* KBL396 and placebo groups (Fig. 6a). There were no significant differences in alpha diversity or beta diversity between the *L. plantarum* KBL396 and placebo groups (Fig. 6b, c). However, the relative abundances of *Lactiplantibacillus* (*p* < 0.0001) and *Akkermansia* (*p* < 0.05) were significantly increased in the *L. plantarum* KBL396 group after the 8 weeks of administration (Fig. 6d).

### Safety

Throughout the study, a total of 34 adverse events (AEs), including 32 mild and two moderate cases, were reported in the *L. plantarum* KBL396 group. Fourteen mild AEs were reported in the placebo group. All AEs potentially related to the *L. plantarum* KBL396 administration were mild gastrointestinal symptoms, which completely resolved without extra intervention. Blood and urine samples were collected from participants at visits 1 and 4 to assess safety. Only eosinophil was significantly lower in the *L. plantarum* KBL396 group compared to the placebo group (*p* < 0.05). Other safety parameters did not show significant differences between the groups.

## Discussion

The gut–brain axis, the bidirectional network connecting gut microbiota to the CNS, can critically regulate mood, cognition, and behavior [1, 36, 37]. A key mechanism in the gut–brain axis is the production of neurotransmitters, such as serotonin (5-HT) and dopamine, which have significant effect on CNS modulation [2, 17, 21, 38]. Tryptophan hydroxylase 1 is the rate-limiting enzyme for 5-HT biosynthesis in the gut [18, 39, 40]. Therefore, we initially focused the effect of *L. plantarum* KBL396 on the *Tph1* gene level of RIN-14B cells, which can directly affect tryptophan hydroxylase 1 expression. Compared to other LAB strains, *L. plantarum* KBL396 significantly upregulated the *Tph1* gene, suggesting its potential to enhance 5-HT synthesis (Fig. 1a). *L. plantarum* KBL396 produced substantial amounts of acetic acid and lactic acid, which are the major short-chain fatty acids (Fig. 1b). This is particularly relevant as gut-derived lactic acid has been shown to influence host 5-HT production and improve anxiety-related behaviors [38]. Moreover, *L. plantarum* KBL396 genome contains various genes for the predicted metabolic pathways related to tryptophan metabolism (Fig. 1c), indicating that *L. plantarum* KBL396 could have an important role in boosting host 5-HT production.

Our in vitro findings translated effectively to our in vivo models. *L. plantarum* KBL396 administration significantly increased serum 5-HT levels in both mice with CSDS and in human participants (Fig. 2f) (Fig. 5a). In mice, the increase of systemic 5-HT levels was accompanied by significant improvements in CSDS-induced depressive-like behaviors. The social interaction ratio was also positively correlated with serum 5-HT levels (Fig. 2b–g). These results strongly suggest that *L. plantarum* KBL396 can induce a systemic increase in the 5-HT level of the host. However, the precise mechanism by which peripheral 5-HT influences CNS function remains still unclear as 5-HT itself does not easily cross the blood–brain barrier. Several mechanisms, including vagal nerve stimulation by enterochromaffin cell-derived 5-HT, microbial regulation of the tryptophan-kynurenine pathway, or peripheral dopamine-mediated neuroimmune modulation, have been proposed [41–43]. Further studies employing precise tracking of gut-derived 5-HT and related neuromodulators are warranted to elucidate the mechanistic basis of *L. plantarum* KBL396 administration on mental health via the microbiota–gut–brain axis.

Figure 3 suggests that percentages of Foxp3^+^CD25^hi^ within CD3^+^CD4^+^ Tregs and CD11c^+^ DCs, which are associated with anti-inflammatory effects [44], were significantly increased in mice with *L. plantarum* KBL396 administration. Our previous studies have revealed that specific strains of *Lactobacillus* spp. have immunomodulatory effects in immune disorders, such as inflammatory bowel disease and atopic dermatitis [45, 46]. These probiotic bacteria can alleviate excessive immune responses via the secretion of anti-inflammatory cytokine interleukin-10 and Foxp3^+^ Tregs. Strong associations between MDD and immune responses have also been reported [47–49]. Therefore, the immunomodulatory effects of *L. plantarum* KBL396 may also play an important role in attenuating mental illness.

Moreover, the 8 weeks of *L. plantarum* KBL396 administration also significantly increased serum 5-HT levels in the habitual drinker (Fig. 5c), a lifestyle factor strongly associated with mental illnesses [50] and has strong positive correlations with MDD [51]. Recent study has suggested that probiotic administration can lead to improvements in depressive symptoms in patients with MDD [52]. *Lactobacillus* administration can also ameliorate gut permeability and tryptophan metabolism via acute stress-induced depression [53, 54]. Therefore, *L. plantarum* KBL396 demonstrates a notable capability in 5-HT biosynthesis and immunomodulation, representing a psychobiotic candidate for supporting mental health.

Furthermore, after the 8 weeks of administration, *L. plantarum* KBL396 increased the relative abundances of *Lactiplantibacillus* and *Akkermansia* in participants. The increase in *Lactiplantibacillus* in the gut indicates successful engraftment of *L. plantarum* KBL396. The concurrent rise in *Akkermansia* is important because it is a keystone taxon recognized for a variety of health benefits, including strengthening host metabolism, enhancing glucagon-like peptide-1 secretion, and improving gut barrier integrity [55, 56]. These findings suggested that *L. plantarum* KBL396 may exert its health benefits not only directly but also by fostering a healthier gut environment through the modulation of gut microbiome. However, further longitudinal studies are needed to monitor these microbial changes over time and to fully elucidate the mechanisms by which *L. plantarum* KBL396 supports mental health.

After the 8-week administration, the *L. plantarum* KBL396 group showed a statistically significant decrease in eosinophils. Eosinophils are leukocytes involved in various immune responses, particularly allergic reactions. However, this finding was not accompanied by changes in other safety markers, including various inflammatory cytokines, and therefore was not considered clinically significant. Other unknown factors, such as specific allergens and participant lifestyles, can also influence eosinophil levels. Therefore, for further applications of *L. plantarum* KBL396 as a novel probiotic for mental health, the associations between major neurotransmitters, such as 5-HT, GABA, and dopamine, and the changes in participant behavior should be assessed via additional human studies with the adjustment of various lifestyle factors and dietary patterns.

By combining mechanistic insights from a validated mouse model with translational findings from a randomized controlled human trial, this study provides a robust framework for evaluating the neuroimmune and microbial effects of *L. plantarum* KBL396. However, this study has several limitations. First, although peripheral 5-HT levels increased following *L. plantarum* KBL396 administration, direct effects on central serotonergic activity remain unconfirmed, as no neuroimaging or brain tissue analysis was performed. Second, while behavioral outcomes were assessed in mice, corresponding psychological evaluations were not included in the clinical trial, limiting translatability. Due to significant physiological and psychological differences between mice and humans, it is challenging to directly extrapolate human outcomes from murine data. Third, the two-to-one randomization, which was applied in this study, could encourage the enrollment and the participation of the participants. However, this model requires a large number of participants for achieving the statistical power for the human study [57]. Therefore, the additional large-scale human studies using the one-to-one randomization method should be performed for further applications of *L. plantarum* KBL396. Fourth, due to the lack of dose–response manner, our study design limitedly suggested the safety and efficacy of *L. plantarum* KBL396. Fifth, the microbiota composition was analyzed taxonomically, but functional profiling (e.g., metagenomics or metabolomics) was not conducted. Finally, despite controlling several lifestyle factors, residual confounding in the human trial cannot be entirely excluded. Therefore, future studies integrated with neurological assessments and multi-omics approaches are needed to fully clarify the psychobiotic mechanisms of *L. plantarum* KBL396.

## Conclusion

*L. plantarum* KBL396 significantly stimulated host 5-HT production in both a preclinical mouse model and a randomized controlled human trial. Moreover, *L. plantarum* KBL396 administration positively modulated host’s gut microbiome with the increases of *Lactiplantibacillus* and *Akkermansia*. These findings preliminarily suggest that *L. plantarum* KBL396 could be used as a psychobiotic candidate for improving mental health via the gut–brain axis–related mechanisms. Further large-scale, longitudinal studies with neurological assessments, phenotypic measurements using SRI or K-BAI score, and multi-omics approaches should be performed to the development of *L. plantarum* KBL396 for the treatment of MDD.

## Data Availability

All data supporting the findings of this study are available from the corresponding author upon reasonable request.
